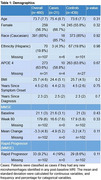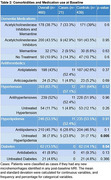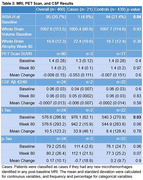# Naturally Occurring ARIA‐H in Alzheimer's disease and Associated Risk Factors: Insights for Clinical Practice

**DOI:** 10.1002/alz70856_101287

**Published:** 2025-12-25

**Authors:** Kristofer Harris, Madison Shyer, Paul E Schulz

**Affiliations:** ^1^ John P. and Kathrine G. McGovern Medical School at UTHealth, Houston, TX, USA; ^2^ UTHealth Houston, Houston, TX, USA

## Abstract

**Background:**

Amyloid related imaging abnormalities‐hemosiderin (ARIA‐H) refers to a form of cerebrovascular alteration observed in Alzheimer's disease (AD) patients, typically linked to anti‐amyloid immunotherapies. However, through placebo controlled clinical trials, we understand that ARIA‐H can also occur naturally in patients with AD, independent of therapeutic interventions. The purpose of this study is to explore associated risk factors for naturally occurring ARIA‐H in patients with AD.

**Methods:**

Using placebo arm data from the EXPEDITION 1 randomized control trial, patients were included in the analysis if they had both baseline volumetric/microhemorrhage MRI data and microhemorrhage MRI data throughout the clinical trial. Patients identified with new ARIA‐H microhemorrhages at any post‐baseline MRI were considered cases. Demographics, comorbidities, medication usage, MMSE, CSF, MRI, and PET scan variables were assessed, and *p*‐values were calculated via Chi‐square, Fisher's exact test, or ANOVA for each variable.

**Results:**

Of the total of 460 patients included in the study, 21 (4.2%, 14 female) patients had incident ARIA‐H and 439 (95.4%, 245 female) were classified as controls. Statistically significant differences between the cases and controls included untreated hyperlipidemia (Cases: 23.8% vs Controls: 7.1%; *p*‐value: 0.01), diabetes diagnosis (Cases: 0.0% vs Controls: 14.1%; *p*‐value: 0.04), ARIA‐H at baseline (Cases: 4.8% vs Controls: 21.4%; *p*‐value: 0.04), and mean CSF t‐Tau at baseline (Cases: 976.1 (SD 62.1) vs Controls: 540.3 (SD 270.9); *p*‐value: 0.03). Traditional anti‐amyloid immunotherapy ARIA‐H risk factors, such as the APOE 4 status and age, did not have a statistically significant *p*‐value. Anticoagulant and antiplatelet use were not associated with incident ARIA‐H.

**Conclusions:**

These findings suggest that modifiable risk factors, such as untreated hyperlipidemia, may contribute to ARIA‐H development. Clinicians should consider prioritizing interventions that reduce the impact of cardiovascular diseases, such as hyperlipidemia, in order to decrease the likelihood of naturally occurring ARIA‐H. Further research is needed using EXPEDITION 2 and 3 data in order to validate these findings, however these initial insights may help guide the clinical decisions moving forward.